# Transforming Microbiological Diagnostics in Nosocomial Lower Respiratory Tract Infections: Innovations Shaping the Future

**DOI:** 10.3390/diagnostics15030265

**Published:** 2025-01-23

**Authors:** Ingrid G. Bustos, Lina F. Martinez-Lemus, Luis Felipe Reyes, Ignacio Martin-Loeches

**Affiliations:** 1 Unisabana Center for Translational Science, School of Medicine, Universidad de La Sabana, Chia 250001, Colombia; ingridbusmo@unisabana.edu.co (I.G.B.); luis.reyes5@unisabana.edu.co (L.F.R.); 2 Clinica Universidad de La Sabana, Chia 250001, Colombia; linaml@clinicaunisabana.edu.co; 3 Pandemic Sciences Institute, University of Oxford, Oxford OX1 2JD, UK; 4 Multidisciplinary Intensive Care Research Organization (MICRO), St James’s Hospital, D08 NHY Dublin, Ireland

**Keywords:** nosocomial respiratory tract infections, microbiological diagnostics, pathogen identification, molecular diagnostics, antimicrobial resistance

## Abstract

**Introduction:** Nosocomial lower respiratory tract infections (nLRTIs), including hospital-acquired pneumonia (HAP) and ventilator-associated pneumonia (VAP), remain significant challenges due to high mortality, morbidity, and healthcare costs. Implementing accurate and timely diagnostic strategies is pivotal for guiding optimized antimicrobial therapy and addressing the growing threat of antimicrobial resistance. **Areas Covered:** This review examines emerging microbiological diagnostic methods for nLRTIs. Although widely utilized, traditional culture-based techniques are hindered by prolonged processing times, limiting their clinical utility in timely decision-making. Advanced molecular tools, such as real-time PCR and multiplex PCR, allow rapid pathogen identification but are constrained by predefined panels. Metagenomic next-generation sequencing (mNGS) provides comprehensive pathogen detection and resistance profiling yet faces cost, complexity, and interpretation challenges. Non-invasive methods, including exhaled breath analysis using electronic nose (e-nose) technology, gene expression profiling, and biomarker detection, hold promise for rapid and bedside diagnostics but require further validation to establish clinical applicability. **Expert Opinion:** Integrating molecular, metagenomic, biomarker-associated, and traditional diagnostics is essential for overcoming limitations. Continued technological refinements and cost reductions will enable broader clinical implementation. These innovations promise to enhance diagnostic accuracy, facilitate targeted therapy, and improve patient outcomes while contributing to global efforts to mitigate antimicrobial resistance.

## 1. Introduction

Nosocomial lower respiratory tract infections (nLRTIs), including hospital-acquired pneumonia (HAP), HAP requiring invasive ventilation (VHAP), ICU-acquired pneumonia (ICU-HAP), ventilator-associated pneumonia (VAP), and ventilator-associated tracheobronchitis (VAT), continue to pose significant challenges in hospital care [1]. Pneumonia accounts for one-third of nosocomial infections in intensive care units (ICUs), with 83% of cases being linked to mechanical ventilation [2]. VAP is particularly concerning, with all-cause mortality ranging from 20% to 50% and a mortality directly caused by this infection attributable to 10% to 13% [1,3,4,5]. HAP, while often less severe, leads to severe complications, such as empyema, septic shock, and multiorgan failure in up to 50% of cases [1,6]. These infections also impose substantial economic burdens, with VAP extending hospital stays by 11 to 13 days and incurring an average of USD 40,000 in excess costs per patient [7,8]. Addressing nLRTIs is thus critical to improving patient outcomes and optimizing healthcare resources.

Given the profound implications for both patient outcomes and the healthcare system, accurate and timely clinical and microbiological diagnoses are essential for guiding appropriate antimicrobial therapy and mitigating the emergence of antibiotic resistance [1,5]. Although widely used, traditional culture-based techniques have significant limitations, particularly in differentiating between infection and colonization. Molecular testing has several advantages over conventional culture-based methods, particularly its ability to detect rapid and precise pathogens. However, molecular methods have limitations, much like culture-based techniques, when distinguishing colonization from infection. Molecular tests, such as real-time PCR or next-generation sequencing (NGS), are highly sensitive and can identify pathogens at lower concentrations, including fastidious or unculturable microorganisms. However, this increased sensitivity is a challenge, as it can lead to the detection of infecting pathogens and colonizing organisms without being able to clearly distinguish between them. This limitation complicates the clinical interpretation of results and highlights the need for complementary diagnostic tools or established thresholds to distinguish between infection and colonization.

To address this limitation, molecular testing can be combined with quantitative thresholds or paired with host-response biomarkers to better assess the clinical relevance of detected pathogens. For example, microbial load measurements or integration with biomarkers like procalcitonin or host gene expression profiles can help provide a more comprehensive picture, distinguishing infection from colonization.

Molecular testing has significant advantages over traditional methods. Unlike culture-based techniques, which are slow and limited by difficulties in detecting fastidious organisms or those suppressed by prior antibiotics, molecular diagnostics provide rapid and sensitive pathogen identification, including resistance markers. By enabling faster, more accurate diagnoses, these methods support earlier targeted therapy, improved antibiotic stewardship, and better clinical outcomes, addressing critical limitations of conventional approaches. Among these innovative approaches, advanced tools such as metagenomic next-generation sequencing (mNGS), matrix-assisted laser desorption/ionization time-of-flight (MALDI-TOF) mass spectrometry, and molecular-biology-based tests, including real-time polymerase chain reaction (qPCR) and multiplex PCR assays, have gained prominence. These technologies substantially improve sensitivity, specificity, and turnaround time, enabling more comprehensive and accurate detection of pathogens causing nLRTIs.

The aim of this review is to comprehensively synthesize evidence-based knowledge regarding microbiological diagnostic methods for nLRTIs. It highlights advancements in traditional techniques, evaluates the expanding role of mNGS and other innovative methodologies, and discusses their potential to revolutionize pathogen detection and clinical decision-making, addressing the critical challenges in contemporary infectious disease management [9].

## 2. Relevant Sections

### 2.1. Overview of nLRTI Definitions

**Figure 1:**
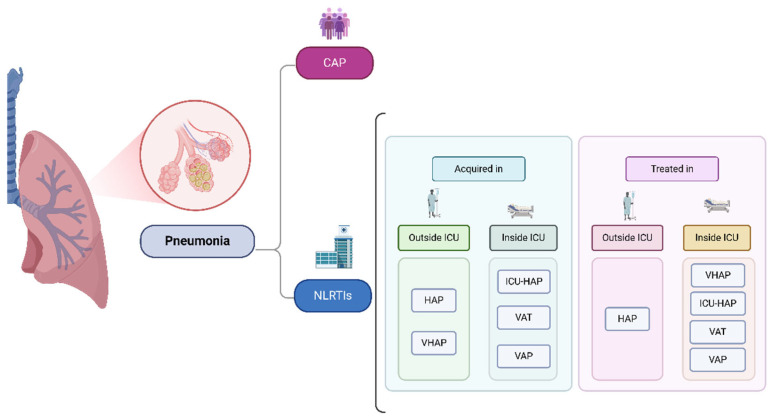
nLRTI classifications: nLRTIs—nosocomial lower respiratory tract infections; HAP—hospital-acquired pneumonia; VHAP—HAP requiring invasive ventilation; ICU-HAP—ICU-acquired pneumonia; VAT—ventilator-associated tracheobronchitis; VAP—ventilator-associated pneumonia.

Standardized definitions of nLRTIs ensure consistent diagnosis and effective management, guiding antimicrobial therapy and patient care. For this review, we used the following clinical criteria: (1) ≥48 h of admission to hospital or ICU, (2) the presence of new or progressive pulmonary infiltrates in chest radiographs (excluding VAT), (3) and at least two of the following indicators: a temperature greater than 38 °C or less than 36 °C; leukocytosis with a volume greater than 12,000 mm^3^ or leukopenia less than 4000 mm^3^ in volume; or the presence of purulent respiratory secretions. In addition, nLRTIs are explicitly defined in Figure 1 and Table 1. Furthermore, we adopted the groups described by the European Network on Respiratory Infections Related to ICUs (ENIRRIs). This classification was chosen because it provides a standardized and robust structure for diagnosis and treatment and considers the complexity of defining nLRTIs in different clinical settings [5,10,11,12].

### 2.2. Etiological Pathogens in nLRTIs

It has been widely reported that nLRTIs are primarily caused by a broad spectrum of bacterial pathogens (with some nLRTIs even being polymicrobial in origin), predominantly driven by a range of Gram-positive and Gram-negative bacteria, with a significant presence of multidrug-resistant (MDR) organisms. Fungal and viral pathogens are less common causes than bacterial pathogens among immunocompetent patients [13]. Moreover, it is recognized that in specific patient populations, such as postoperative patients requiring mechanical ventilation, VAP can be induced by viruses. At the same time, in immunocompromised individuals, infections can be triggered by both viruses and fungi [14].

Recognizing that bacteria constitute the most common cause of nLRTIs, it is essential to highlight that various factors influence the associated pathogens. These include the geographic area in which an infection is acquired, patient-specific characteristics, the length of hospital or ICU stay, the duration of mechanical ventilation in the case of VAP, prior exposure to antimicrobial therapies, risk factors for MDR pathogens, and the local microbial ecology [14,15,16]. Pharyngeal colonization also highlights the interplay between the local microbial ecology and individual risk factors that influence the progression from colonization to infection. For example, community-acquired microorganisms may predominate in non-hospitalized patients, whereas MDR pathogens such as *Pseudomonas aeruginosa* or *Acinetobacter* spp. are more likely to occur in patients with prolonged hospitalization or mechanical ventilation. An understanding of these patterns of colonization can help guide diagnostic and therapeutic strategies, thereby reducing the risk of empirical overtreatment and antimicrobial resistance [17]. Data on HAP etiology mainly come from VAP patients, as endotracheal tubes facilitate collection [16].

**Figure 2:**
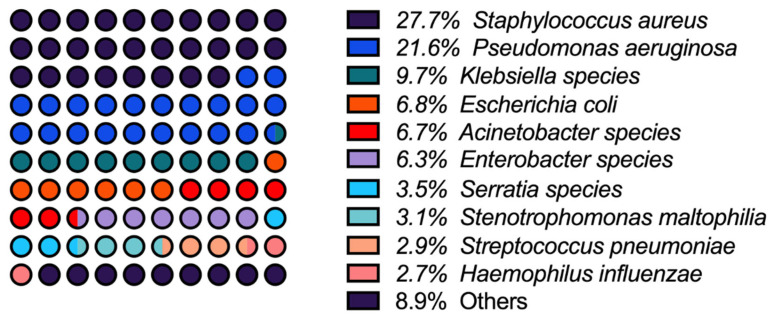
Top pathogens involved in cases of HAP and VAP among patients hospitalized with pneumonia (according to data from the SENTRY Antimicrobial Surveillance Program).

We reviewed various publications and found that a comprehensive review from the journal *Clinical Infectious Disease*—in which data from the SENTRY Antimicrobial Surveillance Program and other supporting studies were analyzed—highlights the most consistent and prevalent pathogens involved in cases of HAP and VAP [18]. According to the findings, the top six pathogens in order of prevalence involved in approximately 80% of episodes are shown in Figure 2 [18]. Despite the previously mentioned low significant difference between the etiologies of HAP and VAP, some results from the SENTRY Program data (2004–2008) highlight substantial differences, such as a higher prevalence of *Pseudomonas aeruginosa* (26.6%) and *Acinetobacter* spp. in VAP patients compared to those with HAP. On the other hand, the incidence of *Staphylococcus aureus* was found to be lower among VAP patients than those with HAP (19.5% vs. 26.6%) [18].

Understanding the microbial causes of nLRTIs is crucial for identifying patients at higher risk of infections caused by difficult-to-treat pathogens, such as MDR [19]. The risk factors have been described by various authors and outlined in the leading international guidelines for the management of HAP and VAP (produced by the Infectious Disease Society of America/the American Thoracic Society [IDSA/ATS] and The European Respiratory Society (ERS), the European Society of Intensive-Care Medicine (ESICM), the European Society of Clinical Microbiology and Infectious Diseases (ESCMID), and Asociación Latinoamericana del Tórax (ALAT) [ERS/ESICM/ESCMID/ALAT]) [1,5]. A summary of these risk factors can be found in Table 2.

Nonbacterial pathogens such as fungi and viruses can contribute to nRLTIs and impact specific patient populations. Among fungi, *Candida species*—the most common yeast found in respiratory samples—colonizes up to 27% of ventilated patients. This colonization may increase the risk of bacterial VAP, particularly that caused by *Pseudomonas aeruginosa.* However, while *Candida* spp. are frequently isolated in respiratory specimens from ventilated patients, they are not considered direct causative agents of VAP. Their presence is typically regarded as colonization rather than infection, and current evidence does not support applying an antifungal treatment solely based on its detection in the respiratory tract [14]. Despite this, Delisle et al. propose that *Candida spp.* colonization is associated with worse clinical outcomes and independently linked to increased hospital mortality [20]. Other fungi implicated in VAP include *Aspergillus species* (notably *Aspergillus fumigatus*), especially for patients with a recent history of influenza infection [14,21]. Finally, respiratory viruses—including influenza, respiratory syncytial virus (RSV), and others—may directly cause VAP. Luyt et al. further suggest that herpes simplex virus (HSV) and cytomegalovirus (CMV) can lead to viral reactivation pneumonia in both immunocompromised and non-immunocompromised mechanically ventilated patients [22].

### 2.3. Microbiological Diagnosis: From Conventional to Advanced Methods

#### 2.3.1. Clinical Samples for Microbiological Diagnosis

The approaches to sample collection for nosocomial lower respiratory tract infections (nLRTIs) reflect a divergence in international guidelines, each prioritizing different strategies based on perceived advantages and contextual considerations. The 2016 ATS/IDSA guidelines emphasize using endotracheal aspirates (ETAs) and semi-quantitative cultures, highlighting simplicity, lower costs, and reduced patient risk [1]. In contrast, the European ERS/ESICM/ESCMID/ALAT guidelines recommend employing quantitative cultures of distal samples obtained through bronchoalveolar lavage (BAL), arguing that these techniques enhance diagnostic accuracy and reduce unnecessary antibiotic use [5].

While the European approach emphasizes diagnostic accuracy, the clinical benefits of these methods, such as a reduced length of stay in the intensive care unit or improved patient outcomes, have not been consistently demonstrated in randomized trials [23,24,25]. Improved diagnostic accuracy does not necessarily translate into measurable improvements in patient care, raising questions about the practical superiority of BAL techniques. Conversely, the ATS/IDSA strategy, although more practical and cost-effective, has inherent trade-offs. These include a higher likelihood of false-positive results due to tracheal colonization or undetected infections, which may compromise clinical decision-making and lead to an inappropriate use of antibiotics [23,26,27].

Both approaches have strengths and limitations that must be critically evaluated in the clinical context. The ATS/IDSA guidelines advocate for a more straightforward, less resource-intensive method that facilitates widespread implementation, especially in settings with limited access to advanced diagnostic tools [1]. However, this approach risks missing critical infections or introducing diagnostic inaccuracies that could adversely affect outcomes. On the other hand, while BAL-based methods offer greater specificity and diagnostic yield, their higher costs, technical demands, and associated risks—such as bronchoscopy-related complications—can limit their feasibility, particularly in resource-constrained environments [24,25,28,29,30].

Our critical evaluation indicates that the decision between these diagnostic approaches should not be made in a dichotomous manner but rather be a customized choice. The selection of diagnostic methods should be informed by factors such as a patient’s clinical condition, the availability of resources, and the local prevalence of antimicrobial resistance. Serial ETA cultures, for instance, offer certain practical advantages, including the capacity to monitor changes in the respiratory microbiota and guide empirical treatment. Nevertheless, their impact on clinical outcomes remains uncertain [31,32,33]. Likewise, non-bronchoscopic bronchoalveolar lavage (BAL) methods, such as mini-BAL or blind aspiration with telescoping catheters, present viable alternatives with comparable sensitivity and specificity within specific contexts, mitigating some inherent limitations of invasive techniques [29]. It is imperative to adopt a balanced approach, integrating the principles of diagnostic accuracy with practical feasibility and patient comfort considerations. Further research is needed to delineate the clinical impact of these strategies, particularly in diverse healthcare settings, to optimize patient outcomes while minimizing risks and resource expenditures [34,35].

#### 2.3.2. Conventional Microbiological Diagnosis Methods

Diagnosing nLRTIs remains a significant challenge, traditionally one in which clinical signs or microbiological diagnostic methods are relied on. In a clinically compatible case, microbiological confirmation of pneumonia involves identifying a pathogen from a lower respiratory tract sample [34]. While this confirmation is valuable, approximately half of pneumonia cases do not have an identifiable causative agent [31]. Traditional methods for the microbiological diagnosis of nLRTIs involve using non-invasive and invasive sampling techniques to obtain samples from the lower respiratory tract, as mentioned previously, followed by Gram stain analysis (in bacterial etiology cases) and culturing. These methods are often employed to differentiate between infection and colonization, as they are less likely to be contaminated by upper-airway flora [13,36].

Gram staining is a rapid diagnostic technique that provides immediate information about the presence and types of bacteria in respiratory samples; for example, it can help predict the presence of *Staphylococcus aureus*, particularly when clusters of Gram-positive cocci are observed. This can guide more personalized antibiotic coverage, although randomized clinical trials are needed to make strong clinical recommendations [37]. However, the correlation between Gram stain results and final culture outcomes can be variable. A meta-analysis found that while Gram stains have a high negative predictive value, their positive predictive value is lower, indicating that a positive Gram stain does not always correlate well with culture results [38]. This suggests that while Gram staining can help rule out infections, it should not be the sole method relied upon to guide antibiotic therapy until culture results are available.

**Figure 3:**
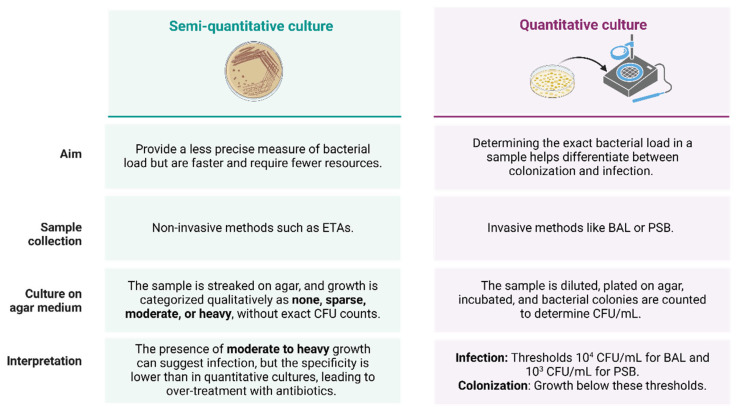
Differences between semi-quantitative and quantitative culturing in the microbiologic diagnosis of nRLTIs.

Quantitative and semi-quantitative culturing are two different approaches used in the microbiological diagnosis of nLRTIs, each with distinct methodologies and implications for clinical practice (Figure 3). Quantitative cultures involve the enumeration of colony-forming units (CFU) per milliliter of sample, providing a precise measure of bacterial load. This method is often used with invasive techniques such as BAL or PSB [1,13]. These cultures have higher specificity, which can help differentiate between colonization and true infections, potentially reducing unnecessary antibiotic use [13,39]. However, they require more laboratory resources and expertise, and their impact on clinical outcomes, such as mortality or length of ICU stay, has not been consistently demonstrated [1].

On the other hand, semi-quantitative cultures categorize bacterial growth into qualitative levels, such as none, sparse, moderate, or heavy, without providing an exact count of CFUs. Such methods are commonly applied to non-invasive samples like endotracheal aspirates. These cultures are faster and require fewer resources, making them more feasible in many clinical settings. While they have high sensitivity and negative predictive value, their specificity is lower than that of quantitative cultures, which can lead to over-treatment with antibiotics [40,41,42].

The leading international guidelines on managing HAP and VAP (IDSA/ATS [1] and ERS/ESICM/ESCMID/ALAT [5]) emphasize the importance of appropriate sampling techniques and the use of local epidemiological data to guide diagnosis and treatment. The 2016 IDSA/ATS Clinical Practice Guidelines recommend using non-invasive sampling methods for HAP and VAP. For ventilated patients, ETAs are preferred. These samples should undergo initial Gram stain and semi-quantitative cultures. Sampling methods using quantitative cultures such as BAL and PSB are not routinely recommended due to the lack of consistent evidence showing improved clinical outcomes [1]. Table 3 summarizes the characteristics of sampling methods used for HAP and VAP. In contrast, the European guidelines, ERS/ESICM/ESCMID/ALAT, suggest obtaining distal quantitative samples before antibiotic treatment since it is known that the results may be altered or emerge as being unfavorable if samples are obtained after starting antibiotic treatment. Both sets of guidelines underscore the importance of differentiating between infection and colonization. The generally accepted thresholds for quantitative cultures are 10^6^ CFU/mL for ETAs, 10^4^ CFU/mL for BAL fluid, and 10^3^ CFU/mL for PSB samples. These thresholds are critical for distinguishing between colonization and infection and aiding in the determination of the appropriate antimicrobial therapy [1,13].

The traditional microbiological diagnosis of nLRTIs presents several challenges, particularly in regard to mixed bacterial species and the risk of unnecessary antibiotic therapy. One significant issue is the polymicrobial nature of respiratory specimens, which complicates the identification of the causative pathogen. Standard culture methods often fail to identify the infectious etiology due to multiple bacterial species and the need for specific tests to detect viral agents [47]. This complexity can lead to empirical antibiotic treatment, which may not be targeted appropriately, increasing the risk of antibiotic resistance and unnecessary exposure to broad-spectrum antibiotics [13,47].

Finally, other conventional microbiologic methods, besides semi-quantitative and quantitative cultures, are used in diagnosing nLRTIs, complementing additional diagnostic information to guide the management of HAP and VAP. One such method is urinary antigen detection, which is particularly useful for detecting specific pathogens such as *Legionella pneumophila* and *Streptococcus pneumoniae.* These tests offer a non-invasive way to identify these organisms rapidly and are highly specific (>90%) for detecting pathogens like *Legionella pneumophila* and *Streptococcus pneumoniae*, though their sensitivity varies. For *S. pneumoniae*, sensitivity ranges from 52% to 86% depending on bacteremia status [13,48]. Turning to blood culturing, Luna et al. highlight that blood cultures have limited value, as most bacterial cases of pneumonia in the ICU are not associated with bacteremia. A positive blood culture may suggest an alternative diagnosis, such as line-associated sepsis [33,49]. Additionally, fungal stains (e.g., KOH with calcofluor), qualitative galactomannan (GM) detection (e.g., The Aspergillus-specific lateral flow device [AspLFD]), and cultures can be performed on respiratory samples, which are helpful for patients at risk of fungal infections, such as those with immunosuppression [13,48,50].

#### 2.3.3. Advances in Molecular and Metagenomic Diagnostics

Advanced microbiological diagnostics in nLRTIs have evolved with the introduction of molecular and metagenomic methods, complementing conventional testing and accelerating the identification of pathogens and resistance markers.

Molecular diagnostic methods are crucial for optimizing antimicrobial therapy, yet conventional culturing methods require 48 to 72 h to yield results, limiting their utility in urgent scenarios [25]. Molecular tests, such as multiplex syndromic panels, have emerged as rapid and accurate tools for detecting intracellular and extracellular pathogens (Table 4). Techniques such as PCR, multiplex PCR, NASBA, and LAMP offer high sensitivity and specificity, enabling direct pathogen identification from clinical samples and overcoming culturing limitations, particularly for fastidious organisms or those under prior antimicrobial treatment [24,51]. Despite their advantages, these tests are complementary rather than replacements for cultures, with limited evidence supporting their impact on clinical outcomes [34].

Initially developed to detect common respiratory viruses and atypical bacteria, molecular diagnostic tools have evolved to encompass a broader range of respiratory pathogens. A prominent example is the FilmArray Pneumonia Plus (FAPP) panel, which has demonstrated a superior ability to detect coinfections compared to conventional methods, which often underestimate the incidence of polymicrobial HAP [25]. These platforms also enable the identification of antimicrobial resistance markers; however, the absence of resistance genes does not guarantee pathogen susceptibility. Undetected resistance mechanisms or resistance genes present in microorganisms below detection thresholds emphasize the necessity of complementing these diagnostics with culture-based techniques in certain cases [25]. Additionally, the over-detection of microbial and viral genomes complicates result interpretation, particularly in terms of distinguishing active pathogens from colonizers. This challenge can be partially addressed through the semi-quantification of bacterial targets.

While molecular diagnostic tools offer numerous advantages, including the ability to simultaneously detect bacterial and viral pathogens through fully automated systems that integrate extraction, amplification, and analysis, they also face limitations. These include the potential for false positives, particularly due to non-viable viruses or viral colonization in asymptomatic patients, genetic variability among pathogens, and the lack of validation studies specific to nosocomial respiratory infections [24]. Moreover, high initial costs and limited availability in certain institutions remain significant barriers. This highlights the need to assess such methods’ impact compared to that of traditional methods, not only in terms of optimizing antimicrobial therapy but also in regard to improving clinical outcomes and combating resistance. Despite advancements in molecular diagnostics, culture-based techniques remain indispensable for complementing and validating molecular findings, particularly in complex clinical scenarios [24,34].

Metagenomic next-generation sequencing (mNGS) is an ideal method for comprehensively detecting bacteria, fungi, and viruses in clinical samples due to its pan-microbial coverage [51]. Advances such as nanopore sequencing have significantly reduced turnaround times to approximately five hours, making them more suitable for clinical use [51]. Recent studies have shown that mNGS can achieve 98.1% accuracy in detecting HAP-associated pathogens, significantly outperforming traditional culture methods [51]. Additionally, mNGS excels in identifying fastidious and rare pathogens, including respiratory viruses with high mortality rates, which are often missed by conventional methods [51].

Another notable advantage of mNGS is its increasing cost-effectiveness. Library multiplexing and batch sequencing have reduced costs to approximately USD 92 per test compared to traditional methods, which require multiple complementary tests [51]. However, clinical interpretation and decision-making are complicated because these methods’ high sensitivity often results in the detection of colonizers or contaminants. Unlike traditional quantitative cultures, mNGS results are typically reported as relative abundances, providing colony-forming unit (CFU) thresholds with which to distinguish infection from colonization. This lack of standardized thresholds makes it difficult to determine the clinical significance of the detected organisms [24,34]. In addition, mNGS often identifies multiple microbial species, some of which may represent normal respiratory flora or environmental contaminants rather than true pathogens [52,53].

In addition, the lack of standardized thresholds for microbial load in mNGS results increases the complexity of interpretation. Unlike traditional quantitative cultures, mNGS results are typically reported as the relative abundance of organisms detected, which provides clear colony-forming unit (CFU) thresholds with which to distinguish colonization from infection. This lack of standardization can lead to inconsistent clinical application and variable interpretations between institutions [54].

A hybrid approach that integrates mNGS with traditional culture-based methods is recommended to maximize the clinical utility of mNGS while minimizing its limitations. Traditional cultures remain essential to provide CFU-based quantification. Currently, mNGS lacks these elements. In this combined strategy, mNGS can serve as a complementary diagnostic tool, providing rapid broad-spectrum detection, while culture methods validate findings and guide treatment decisions [55].

Future research should establish standardized protocols for mNGS data interpretation, including thresholds for differentiating pathogens from colonizers and contaminants [54]. This is particularly critical given the challenges of interpreting molecular and metagenomic results, such as distinguishing colonization from infection, differentiating viable from non-viable microorganisms, and understanding their significance within the microbiome context [56]. Incorporating machine learning algorithms, as suggested by Beam and Kohane [57], may further enhance diagnostic accuracy and reduce interpretative ambiguity, ultimately positioning mNGS as a valuable tool for clinical practice, especially in complex cases requiring comprehensive pathogen detection [24,34].

#### 2.3.4. Biomarkers and Proteomics in the Diagnosis of nLTRIs

In the clinical management of infections, including nosocomial lower respiratory tract infections (nLRTIs), biomarkers such as C-reactive protein (CRP), procalcitonin (PCT), and soluble triggering receptor expressed on myeloid cells (sTREM-1) play an important role. Their integration into routine practice remains variable, although their diagnostic utility and clinical adoption have shown promise in improving antimicrobial stewardship and decision-making. This variability is influenced by clinical context, patient population, and the need for the standardization of biomarker use. These challenges underscore the importance of validating biomarkers to improve their consistency and reliability in various healthcare settings [58,59,60]. CRP trends, when combined with a clinical assessment, help monitor infection progression or treatment response for critically ill patients [61,62]. However, their limited specificity makes them less reliable for differentiating bacterial and viral infections. PCT offers greater specificity for bacterial infections and supports antibiotic decision-making in ventilator-associated pneumonia (VAP) and sepsis [63]. This reduces unnecessary antibiotic use without compromising outcomes [64]. These biomarkers are particularly valuable in resource-limited settings, guiding escalation/de-escalation based on trends and clinical signs [65,66]. Evaluations of protein-based biomarkers—including both pathogen-specific and host-response markers—offer valuable insights into their diagnostic relevance [59]. The evaluation of serial measurements over time is critical to ensure the safety and reliability of clinical decisions based on these markers. This approach allows more precise and informed adjustments to be made to management strategies by providing dynamic insights into patient responses [59].

Advancements in genomics, transcriptomics, proteomics, and metabolomics underscore the potential of omics technologies in identifying host–pathogen interaction signatures [59]. These technologies support the prediction, diagnosis, and prognosis of infections. Multimarker models, combining several biomarkers, have demonstrated greater efficacy than single biomarkers for disease risk assessment [60]. For example, combining IL-1β, IL-8, MMP-8, MMP-9, and NHE in BAL samples has shown diagnostic potential [67]. The Bioscore model—incorporating seven biomarkers from BAL fluid and serum, such as the TREM-1 BALF/blood ratio, sTREM-1, IL-8, IL-1β, CRP, and IL-6—correctly identified 88.9% of VAP cases and 100% of non-VAP cases [59]. These approaches have promise for early antibiotic intervention in VAP.

Proteomic analysis of BAL fluid further refines the diagnosis of acute lung injury (ALI) with or without VAP. In VAP-positive patients, BAL fluid is enriched with proteins involved in inflammation, immune defense, and immunity, including ITGB2, ITGAM, and myeloperoxidase (MPO), which facilitate neutrophil adhesion, transmigration, and bacterial phagocytosis [68]. In contrast, VAP-negative patients show higher levels of proteins related to wound healing and tissue repair, such as fibronectin 1 (FN1), which is downregulated in VAP-positive cases [68]. These findings indicate a pathological imbalance in VAP, characterized by an excessive quantity of pro-inflammatory mediators and reduced reparative protein levels [68]. Computational tools integrated into BAL proteomics hold potential for classifying pulmonary disorders and understanding disease mechanisms.

Studies evaluating panels of biomarkers—including cytokines and oxylipins—have effectively differentiated ICU patients with pneumonia from those with lung injury [58]. Elevated levels of E-selectin, MCP-1, ICAM-1, and IP-10 distinguish pneumonia from brain injury [58]. In VAP cases, increased levels of IL-6, IL-8, and ICAM-1 reflect an inflammatory response to infection [58]. These panels can also identify brain injury patients who develop VAP [58]. Biomarkers thus play a complementary role in diagnosing pulmonary infections and tailoring antimicrobial therapy [59].

Although the clinical use of biomarkers has increased, robust evidence supporting their predictive reliability is still needed [60]. Single-protein biomarkers have shown inconsistent results in predicting VAP onset or severity [60]. Therefore, a translational approach combining genomic, proteomic, and metabolomic methodologies is crucial to advancing our understanding of these complex infections [60].

#### 2.3.5. Integration of Gene Expression Profiles in the Diagnosis of nLTRI

Genomic detection methods play a pivotal role in identifying genetic factors and gene expression profiles associated with the development of nLTRIs [69]. These profiles can distinguish between patients who develop an infection and those who do not, enabling the prediction of infection onset before clinical symptoms appear [70]. High-throughput technologies, such as microarray analysis, facilitate the identification of differentially expressed genes and provide insights into the complex responses of hosts to infection [71,72].

In ventilator-associated pneumonia (VAP), distinct gene expression profiles have been identified, showing downregulation of genes like PIK3R3, ATP2A1, PI3, ADAM8, and HCN4 [69]. This downregulation suggests impaired immune response, muscle dysfunction, and physiological dysregulation, making patients more susceptible to VAP following LPS stimulation [69]. In contrast, genes such as ELANE, LTF, and MAPK14 are overexpressed, indicating dysregulated activation of the MAPK signaling pathway, specifically p38 MAPK, which may heighten infection susceptibility [72].

In hospital-acquired pneumonia (HAP), overexpression of genes related to cell–cell junction remodeling, adhesion, and leukocyte diapedesis has been observed [73]. Concurrently, the downregulation of genes involved in type I interferon signaling weakens antibacterial defenses, increasing vulnerability to less virulent pathogens [73]. Additionally, reduced levels of MMP-8 and soluble E-selectin suggest enhanced leukocyte adhesion and endothelial migration, promoting systemic inflammation [73]. These profiles not only differentiate between inflammation and infection [71] but also provide a foundation for developing predictive diagnostic tools and patient stratification strategies in ICUs.

Recent transcriptomic studies using microarrays have identified gene signatures in patients with VA-LRTI within 24 h of diagnosis [74]. These studies employed AUROC analysis to assess mRNA levels’ diagnostic accuracy. Although significant overlap in gene expression levels was observed between VAP and VAT patients—particularly in genes such as HLA, IL2RA, CD40LG, ICOS, CCR7, CD1C, and CD3E—both groups exhibited immunosuppression, indicative of immune paralysis [74]. However, VAP patients displayed more pronounced immune dysfunction, potentially explaining the progression from VAT to VAP [74].

Combining gene expression profiles with advanced technologies like artificial intelligence (AI) and next-generation sequencing (NGS) can refine the early diagnosis of VA-LRTI, enhance treatment monitoring, and enable precise patient stratification in ICUs [74]. Continued research integrating multiple gene signatures and transcriptional markers is essential for optimizing nLTRI diagnosis and management [72,74].

#### 2.3.6. Other Innovative Microbiological Diagnostic Methods for nLRTIs

Exhaled-breath analysis has emerged as an innovative, non-invasive technology for microbiological diagnostics in HAP and other respiratory infections [75]. This approach relies on detecting volatile organic compounds (VOCs), metabolic byproducts generated by microorganisms through pathways such as glycolysis, proteolysis, and lipolysis [2]. These innovative tools must be rapid, user-friendly, and effective in supporting critical clinical decisions [76].

A promising technology in this field is the electronic nose (e-nose), which uses sensors to identify specific VOC patterns associated with various microorganisms. The data obtained are processed through machine learning algorithms to develop diagnostic prediction models [2]. Studies have demonstrated that e-noses can differentiate pathogens such as *Staphylococcus aureus*, *Escherichia coli*, *Candida albicans*, and *Acinetobacter baumannii*, suggesting its potential for developing non-invasive therapies [2]. Additionally, in vitro investigations have confirmed the e-nose’s ability to identify bacteria like *Haemophilus influenzae*, *Pseudomonas aeruginosa*, and *Streptococcus pneumoniae* [2].

Exhaled-breath analysis has also been combined with machine learning techniques. These algorithms analyze VOC data to construct predictive models capable of distinguishing viral infections from bacterial ones, as demonstrated in a pilot study involving patients with acute COPD exacerbations [76]. However, factors such as comorbidities (e.g., diabetes, acute renal failure, and hepatitis) and multiple infections may affect the diagnostic accuracy of these devices [2].

Despite current limitations, such as insufficient diagnostic specificity for VAP patients, the e-nose offers significant practical advantages, including portability and rapid detection capacity. Furthermore, integrating the e-nose alongside tools like gas chromatography and mass spectrometry could identify specific biomarkers in exhaled breath, improving the device’s sensitivity and specificity for clinical applications [75].

Finally, exhaled-breath analysis is a non-invasive method that distinguishes between healthy and diseased individuals based on VOC composition. While some studies report good sensitivity and specificity for lower respiratory tract infections, diagnostic accuracy remains insufficient and requires further validation [24]. A recent study identified target gases such as carbide, sulfide, and ammonia for detecting VAP, highlighting the potential of these innovative approaches in microbiological diagnostics [24].

## 3. Expert Opinion

The rapid and accurate identification of pathogens in nLRTIs plays a crucial role in guiding antimicrobial therapy. Timely pathogen identification can support targeted treatment decisions and reduce reliance on empirical therapy, while appropriate antimicrobial therapy is the primary determinant of improved clinical outcomes. However, fixed timeframes for specimen collection, processing, and analysis are currently required for microbiological techniques. These delays can in turn delay the initiation of appropriate treatment and, in some cases, increase the risk of inappropriate antibiotic administration. This can affect patient outcomes [77].

The complexity of nLRTIs, coupled with the growing number of hospitalized patients colonized by or infected with resistant pathogens, has led clinicians to lower their threshold for prescribing broad-spectrum empirical antibiotics. Although this practice is intended to protect the most vulnerable patients, prior antibiotic exposure is a significant risk factor for ineffective antimicrobial treatment. In these cases, the infrequency of confirmed microbiological diagnoses underscores the need for a pragmatic diagnostic approach that balances speed and accuracy.

Diagnostic techniques have evolved to address these challenges by reducing response times and providing more detailed information. Molecular methods, such as PCR, deliver results within hours, which can be compared to the days required by traditional cultures. Nevertheless, their clinical application remains limited to predefined pathogen panels and research settings. Additionally, these techniques struggle to differentiate between colonization and active infection, which restricts their utility in specific clinical contexts.

Metagenomic next-generation sequencing (mNGS) serves as a promising alternative by analyzing all nucleic acids present in a sample, enabling the identification of a broad range of pathogens, including difficult-to-culture organisms such as Mycobacterium tuberculosis, *Legionella* spp., viruses, and fungi. This approach overcomes some of the limitations of PCR by not relying on specific targets, but interpreting its results is challenging due to the possibility of detecting normal microbiota, colonizers, or contaminants. In bronchoalveolar lavage fluid (BALF) samples, this complexity is exacerbated, as the presence of a microorganism does not necessarily indicate active infection. The lack of standardized interpretation guidelines further complicates its use, introducing variability and subjective biases that hinder consistency across institutions. Furthermore, despite recent advances, the high cost, need for skilled personnel, and extensive bioinformatics infrastructure required mean that mNGS remains primarily limited to specialized reference laboratories. To maximize its clinical utility and ensure wider adoption, it is essential to address these limitations through standardized protocols and integration with traditional methods.

Non-invasive technologies, such as exhaled-breath analysis using devices like the electronic nose (e-nose), also present a promising diagnostic approach. These methods offer rapid, bedside-usable diagnostics by identifying pathogen-specific patterns of volatile organic compounds (VOCs). However, their accuracy may be compromised by factors such as patient comorbidities and polymicrobial infections, necessitating further technological refinement and clinical validation before widespread adoption.

The diagnosis of nLTRIs using biomarkers and gene expression profiles holds significant potential, though its clinical application remains challenging. Biomarkers such as CRP, procalcitonin, and sTREM-1 provide valuable insights, but variability in results limits their widespread adoption. The identification of differentially expressed genes like PIK3R3, ATP2A1, and HLA-DRA offers a detailed understanding of immune suppression in critically ill patients. However, the lack of standardized protocols and the complexity of data interpretation hinder clinical implementation. A multimodal approach that integrates proteomic biomarkers, transcriptomics, and advanced tools such as artificial intelligence (AI) could enhance diagnostic accuracy and optimize antimicrobial therapy. Combining these technologies is crucial for early detection, precise patient stratification, and improved clinical outcomes for nLTRI patients.

In conclusion, although current diagnostic technologies offer significant advantages in terms of speed and precision, practical and technical limitations remain major challenges. Integrating molecular, metagenomic, and traditional diagnostic methods is essential to optimize the management of nLRTIs. Optimizing, refining, standardizing, and reducing the costs of these technologies are essential for wider clinical implementation. Such advances will improve patient outcomes and play a key role in the fight against antimicrobial resistance. They will also ensure that diagnostic precision matches therapeutic efficacy, leading to more targeted and effective antimicrobial treatments.

## 4. Future Directions

Reducing the time between sample collection and precise pathogen identification remains a critical challenge in managing non-lower respiratory tract infections (nLRTIs). Traditional microbiological methods, which typically require 48 to 72 h to identify pathogens and determine antimicrobial susceptibility profiles, hinder timely therapeutic decision-making. This delay can lead to prolonged use of broad-spectrum antibiotics, increasing the risk of adverse effects such as intestinal colonization by resistant bacteria or *Clostridium difficile* infections. Moreover, in cases of suspected ventilator-associated pneumonia, relying solely on these delayed methods may postpone appropriate treatment or result in the administration of ineffective antibiotics, which have both been associated with higher mortality rates.

Emerging technologies offer promising solutions to these challenges. Molecular techniques such as real-time PCR have significantly reduced diagnostic times compared to culture-based methods, enabling pathogen identification within hours. However, the potential of metagenomic next-generation sequencing (mNGS) to analyze the entire spectrum of nucleic acids in a sample presents an opportunity to overcome the limitations of PCR. mNGS can identify various pathogens, including difficult-to-culture organisms, and provide valuable insights into antimicrobial resistance markers and virulence factors. Although currently restricted to reference laboratories due to high costs and technical requirements, sequencing speed and cost-efficiency advances could make mNGS a routine clinical tool. Achieving laboratory turnaround times of 24 h—or even 6 h in research settings—suggests that further developments could revolutionize clinical diagnostics.

Non-invasive diagnostic methods, such as exhaled-breath analysis using electronic nose (e-nose) devices, represent another frontier with the potential for rapid, bedside diagnostics. These tools analyze pathogen-specific volatile organic compounds (VOCs) and could complement molecular and metagenomic approaches. Although accuracy remains a concern due to patient comorbidities and polymicrobial infections, continued refinement and validation of e-nose technology may facilitate its adoption into clinical practice.

Emerging AI technologies, particularly machine learning algorithms, are showing promise in predicting infection risk, enhancing early detection, and personalizing treatment strategies. By analyzing vast datasets—including patient demographics, vital signs, and laboratory results—AI can identify subtle patterns that may precede clinical symptoms. As AI models continue to evolve, integrating these systems with clinical workflows could enable real-time decision-making, optimize the use of antimicrobial therapies, and reduce diagnostic delays. Moving forward, developing standardized protocols, ensuring data interoperability, and validating AI models across diverse patient populations will be essential. These advancements could significantly improve patient outcomes, reduce morbidity and mortality, and play a crucial role in combating antimicrobial resistance.

The future of nLRTI diagnostics lies in incorporating these innovations to address current limitations. Combining rapid molecular techniques, comprehensive metagenomic analyses, and non-invasive methods with insights from biomarkers and gene expression profiles could transform clinical decision-making by providing faster and more accurate results. This integration would reduce reliance on broad-spectrum antibiotics, promote targeted therapy, and enhance antimicrobial stewardship. As these technologies evolve and become more accessible, they hold the potential to improve patient outcomes and strengthen global efforts to combat antimicrobial resistance.

## Figures and Tables

**Table 1:** nLTRI classifications.

nLTRI Classifications	Definition
HAP	LTRIs acquired outside the ICU at least ≥ 48 h after admission that do not require invasive mechanical ventilation
VHAP	LRTIs acquired outside the ICU at least ≥ 48 h after admission that require invasive mechanical ventilation
ICU-HAP	LRTI acquired at least ≥ 48 h after ICU admission that do not require invasive mechanical ventilation
VAP	A condition in which a patient who is admitted to the ICU requiring mechanical ventilation for reasons different from LRTI and, after ≥ 48 h of tracheal intubation/tracheostomy, develops an LRTI
VAT	A condition in which a patient who is admitted to the ICU requiring mechanical ventilation for reasons different from LRTI develops an LRTI at least ≥ 48 h after tracheal intubation/tracheostomy without a new or progressive radiological pulmonary infiltrate being detected

**Table 2:** High-MDR-risk pathogens in HAP/VAP.

European Guidelines (ERS/ESICM/ESCMID/ALAT) [5]	American Guidelines (IDSA/ATS) [1]
High-Risk HAP/VAP Septic shock and/or the following risk factors for potentially resistant microorganisms: Hospital settings with rates of MDR pathogens ≥25%, including Gram-negative bacteria and MRSA Previous antibiotic use Recent prolonged hospital stay (≥ 5 days of hospitalization) Previous colonization with MDR pathogens	Risk factors for MDR HAP Prior intravenous antibiotic use within 90 days Risk factors for MDR VAP Prior intravenous antibiotic use within 90 days Septic shock at the time of VAP Acute respiratory distress syndrome (ARDS) preceding VAP Five or more days of hospitalization prior to the occurrence of VAP Requirement for acute renal replacement therapy before VAP onset Risk factors for MRSA VAP/HAP Prior intravenous antibiotic use within 90 days Risk factors for MDR Pseudomonas VAP/HAP Prior intravenous antibiotic use within 90 days

**Table 3:** Sampling methods for HAP and VAP.

Sampling Method	Advantages	Disadvantages	References
Non-Invasive Sampling Methods			
Spontaneous Expectoration	Easy to perform	Lower diagnostic accuracy due to contamination with oral and nasopharyngeal flora	[13]
Sputum Induction	Useful for non-intubated patients, can improve diagnostic yield	Requires patient cooperation, poses risk of inducing bronchospasm, potential for contamination with upper respiratory flora	[13,43]
Nasotracheal Suction	Useful for uncooperative patients	Risk of contamination with upper-respiratory flora, lower diagnostic accuracy	[13]
Endotracheal Aspirate (ETA)	Rapid, less costly, less invasive, easy to perform, high sensitivity (75.7%)	Lower specificity (67.9%), risk of contamination with upper-respiratory flora	[13,44]
**Invasive Sampling Methods**			
Bronchoalveolar Lavage (BAL)	Higher specificity (79.6%) and sensitivity (71.1%), better for differentiating colonization from infection	More costly, requires trained personnel and specialized equipment, poses risk of complications like hypoxemia and bleeding	[1,36,44]
Protected Specimen Brush (PSB)	High specificity (76.5%), minimizes contamination from upper respiratory tract	Lower sensitivity (61.4%), more invasive and costly, requires bronchoscopy	[1,36,44]
Blind Bronchial Sampling	Can be performed without bronchoscopy, useful in settings in which bronchoscopy cannot be conducted, good diagnostic yield	Poses risk of contamination, lower specificity compared to bronchoscopic methods, requires skill to perform correctly	[1,45,46]

**Table 4:** Molecular techniques for the simultaneous detection of microorganisms in respiratory samples.

Commercial Name	Technique Used	Year of Launch
CLART ^®^ PneumoVir	Multiplex Real-Time and Microarrays	2008
Prodesse ProFLu+ Assay	Multiplex Real-Time PCR	2008
xMAP ^®^ Respiratory VIral Panel	Bead-Based Arrays	2008
Luminex xTAG^®^ Respiratory Viral Panel	Multiplex PCR and Bead-Based Array	2008
GenoSensor ^®^ Respiratory Virus Panel	Microarrays	2009
RespiFinder ^®^ 22	Multiplex Real-Time PCR	2010
RespiFInder ^®^ 2Smart	Multiplex Real-Time PCR	2010
FilmArray ^®^ Respiratory Panel	Multiplex Real-Time PCR	2011
FTD Respiratory Pathogens 21	Multiplex Real-Time PCR	2011
Simplexa^TM^ Respiratory Panel	Multiplex Real-Time PCR	2012
AdvanSureTM RV Real-Time RT-PCR Kit	Multiplex Real-Time PCR	2013
Anyplex^TM^ II RV16 Detection	Multiplex Real-Time PCR	2013
PowerChek^TM^ Respiratory Viral Real-Time PCR Kit	Multiplex Real-Time PCR	2014
Verigene ^®^ Respiratory Pathogens Flex Test	Multiplex Real-Time PCR	2014
NxTAG ^®^ Respiratory Pathogen Panel	Multiplex Real-Time PCR	2015
RIDA ^®^ GENE Respiratory Panel	Multiplex Real-Time PCR	2015
15Allplex ^TM^ Respiratory Panel	Multiplex Real-Time PCR	2016
ePlex ^®^ Respiratory Pathogen Panel	Multiplex Real-Time PCR	2017
QIAstat-Dx ^®^ Respiratory Panel	Multiplex Real-Time PCR	2018
BioCode ^®^ Respiratory Pathogen Panel	Multiplex Real-Time PCR	2019

## Data Availability

Not applicable.
